# Drugging the Small GTPase Pathways in Cancer Treatment: Promises and Challenges

**DOI:** 10.3390/cells8030255

**Published:** 2019-03-16

**Authors:** Néstor Prieto-Dominguez, Christopher Parnell, Yong Teng

**Affiliations:** 1 Department of Oral Biology and Diagnostic Sciences, Dental College of Georgia, Augusta University, Augusta, GA 30912, USA; nprid@unileon.es; 2 Institute of Biomedicine (IBIOMED), University of León, 24010 León, Spain; 3 Medical College of Georgia, Augusta University, Augusta, GA 30912, USA; CPARNELL@augusta.edu; 4 Department of Medical laboratory, Imaging and Radiologic Sciences, College of Allied Health, Augusta University, Augusta, GA 30912, USA

**Keywords:** small GTPases, Arf1, inhibitors, cancer therapy, anticancer

## Abstract

Small GTPases are a family of low molecular weight GTP-hydrolyzing enzymes that cycle between an inactive state when bound to GDP and an active state when associated to GTP. Small GTPases regulate key cellular processes (e.g., cell differentiation, proliferation, and motility) as well as subcellular events (e.g., vesicle trafficking), making them key participants in a great array of pathophysiological processes. Indeed, the dysfunction and deregulation of certain small GTPases, such as the members of the Ras and Arf subfamilies, have been related with the promotion and progression of cancer. Therefore, the development of inhibitors that target dysfunctional small GTPases could represent a potential therapeutic strategy for cancer treatment. This review covers the basic biochemical mechanisms and the diverse functions of small GTPases in cancer. We also discuss the strategies and challenges of inhibiting the activity of these enzymes and delve into new approaches that offer opportunities to target them in cancer therapy.

## 1. Introduction

Small GTPases are a large family of hydrolases that bind and hydrolyze GTP to GDP in order to regulate many cellular activities (e.g., cell differentiation, proliferation, and motility) [1]. Inherently, small GTPases have a basal mild endogenous GTPase activity that is dependent on Mg^2+^ to weaken the bond between the last two phosphates in GTP in order to form GDP [2,3,4]. Small GTPases are normally maintained in an GDP-bound inactive state and are induced by guanine-nucleotide exchange factors (GEFs), which replace the GDP molecule that normally occupies the small GTPase binding pocket with GTP [4]. Equally important for the regulation of small GTPases are the GTPase-activating proteins (GAPs), which deactivate small GTPases through enhancing their endogenous hydrolytic activity, leading to the shifting of GTP to GDP and their subsequent inactivation [4].

Small GTPases have been grouped into five major classes according to their sequence homology and on their physiological functions [3]: Arf subfamily, Ras subfamily, Ras-homolog (Rho) subfamily, Ras-related in brain (Rab) subfamily, and Ras-related nuclear protein (Ran) subfamily [5,6]. However, the Ran and the Rab subfamilies have recently been fused due to the high homology that presents their components [7].

**Figure 1:**
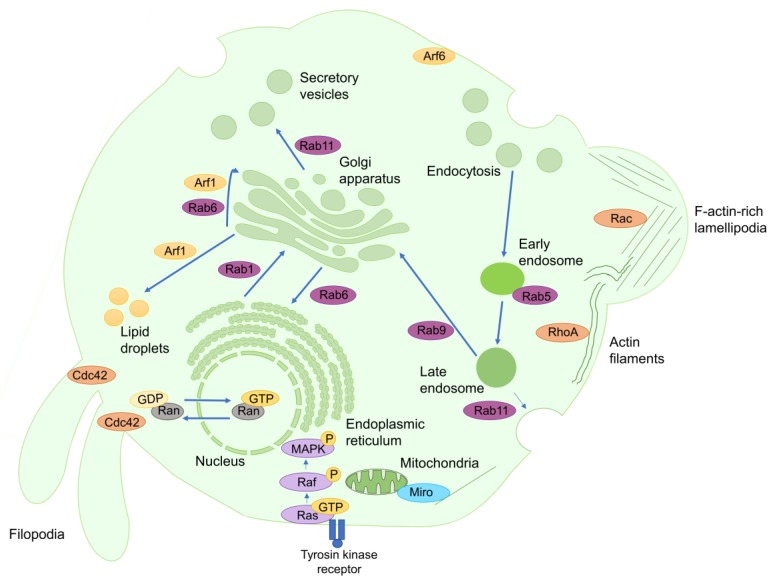
Role of small GTPases in human cells. Most small GTPases are implied in the regulation of protein secretion, endocytosis and vesicle trafficking. For instance, Ran-activation gradient controls both the export and import of macromolecules between the nucleus and the cytoplasm. Additionally, Rab1 is responsible for regulation of vesicle trafficking between the endoplasmic reticulum and the Golgi apparatus, whereas Rab6 modulates the reverse transport, as well as through the different Golgi apparatus vesicles. Arf1 is implied in intra-Golgi transport, but also enables the accumulation of fatty acids inside the lipid droplets. Otherwise, Rab5 regulates endosome coating. The control of secretory vesicle formation is mainly mediated by Rab11. The products resulting from phagosome digestion can be carried to Golgi apparatus in a Rab9-dependent process, or return to the extracellular matrix in a Rab11-dependent mechanism. Arf6, which is associated with the plasma membrane when inactive, works as a master regulator of vesicle processes. On the other hand, other small GTPases are involved in the maintenance of cell shape and movement, such as Rac, which promotes the generation of lamellipodia, or Cdc42, which promotes the formation of filopodia. RhoA induces the formation of actin filaments in response to cellular stresses. Otherwise, Ras induces the phosphorylation and activation of MAPK, inducing prosurvival responses, such as cell proliferation and cell cycle progression, as well as limiting prodeath signals, such as apoptosis.

The Arf subfamily is involved in a broad spectrum of physiological roles, such as the organization of the cytoskeleton, the sorting of vesicle cargo, the recruitment of vesicle coat proteins, and the alteration of lipid membranes through the recruitment of key enzymes, including phosphatidylinositol-four-phosphate adapter protein 1 (FAPP1), FAPP2, and the ceramide transfer protein (CERT) (Figure 1) [8,9]. The Arf subfamily consists of 6 Arf isoforms, 22 different Arf-like proteins (ARL) and Sar1 [8,10]. Deregulation of some Arf isoforms has been shown to induce cancer formation and progression by enhancing cell proliferation through the activation of mitogen-activated protein kinases (MAPK) and ribosomal protein S6 kinase beta-1 (p70S6K) [11,12]. Furthermore, deregulation of certain Arf family members, such as Arf1 and Arf6, enhances cancer cell invasion and metastasis by stimulating Ras-related C3 botulinum toxin substrate 1 (Rac1), paxillin, talin or focal adhesion kinase (FAK) [12,13,14].

The Ras subfamily includes 36 different members divided into seven subgroups: Ras proteins, Ras-related proteins (RRAS), Ras-like proteins (Ral), Ras-proximal proteins (Rap), Ras-related associated with diabetes (Rad) and Gem-related proteins (GRE), Ras homolog enriched in brain (Rheb), and Ras-like in all tissues proteins (Rit) [7,15]. The members of the Ras subfamily are involved in the activation of intracellular signaling networks, resulting in enhancing cellular proliferation, adhesion, migration, and survival, as well as in limiting apoptosis (Figure 1) [15]. Ras overexpression has been found in more than 15% of human tumors [15], since its upregulation can promote cancer cell proliferation by the deregulation of the basal activation levels of the MAPK, phosphatidylinositol 3-kinase (PI3K), and phospholipase C epsilon (PLCε) pathways [15]. In addition, Ral overexpression stimulates tumorigenesis and tumor invasion in a Ras-dependent manner due to its ability to induce cell exocytosis by interacting with Sec5 and the exocyst complex component 84 (Exo84) [16]. The upregulation of certain Rap members can also promote cancer cell proliferation, migration and invasion due to their capability to alter integrin-mediated cell adhesion [17,18]. Rheb proteins promote carcinogenic cell proliferation and tumorigenesis by promoting the activation of the mechanistic target of rapamycin complex 1 (mTORC1) [19]. Finally, Rit proteins enhance neuronal survival and differentiation, thus their deregulation could be associated with neuronal tumor progression and with the onset of many nervous system pathologies, such as Parkinson’s disease, autism, or schizophrenia [20].

The Rho family consists of 22 proteins divided into seven subgroups: Ras-related C3 botulinum toxin substrate (Rac), Rho-related proteins (RhoA), cell division control protein 42 homolog (Cdc42), TC10 and T-cell leukemia/lymphoma protein 1A (TCL), Rho-related GTP-binding protein Rho6 precursor (Rnd), Rho-related BTB domain-containing protein (RhoBTB) and [21,22]. The members of the Rho family are involved in controlling actin turnover and in coordinating cell shape and movement through the regulation of the activity of the actomyosin complex (Figure 1) [23]. More in detail, Rho subfamily proteins can generate different actin structures to allow the displacement of the cell in response to several mechanical stimuli, such as Rac-dependent filamentous-actin-rich lamellipodia or Cdc42-dependent filopodia protrusions (Figure 1) [21,22]. On the other hand, the aberrant expression of several small GTPases of this family, such as Rac, Cdc42 and RhoA, in carcinogenic cells enhances cancer promotion and progression by facilitating cell cycle progression and mitosis, disrupting tight cellular junctions that prevent cell mobility and inducing epithelial to mesenchymal transition (EMT) to enhance the formation of secondary tumors [21,22]. Moreover, RhoA and RhoC can also promote the formation of new vessels towards the carcinogenic mass through inducing the release of several proangiogenic factors, such as vascular endothelial grown factor [21].

The Rab family consists of approximately 70 members whose main function is to manage vesicular trafficking between intracellular organelles (Figure 1) [24]. The Rab family members select and collect vesicle cargo by increasing the affinity of certain protein sorting receptors, such as the mannose-6-phosphate receptor (M6PR), to the nascent vesicle (Figure 1) [25]. These small GTPases modulate vesicle transport through actin filaments and microtubules by recruiting, respectively, myosin V and kinesins (Figure 1) [25]. Additionally, they induce vesicle fusion by interacting with certain members of the NSF-attachment protein receptors (SNAREs) family that tether the transport vacuole to the acceptor membrane (Figure 1) [25]. The upregulation of certain members of the Rab family (such as Rab25, Rab5 and Rab11) in carcinogenic cells induces tumorigenesis by increasing cell proliferation and migration, via the activation of the Akt/mTORC1, extracellular signal-regulated kinase 1 (ERK1), and Wnt/β-catenin pathways, as well as inhibits tumor cell apoptosis via the decrease of Bak and Bax expression [24,26].

Ran controls molecular export and import from the nucleus to the cytoplasm. Ran GEFs, which activate Ran, accumulate in the nucleus and their interaction with Ran allows the binding and transfer of the cargo from the nucleus to the cytoplasm (Figure 1) [27]. Once in the cytoplasm, the active Ran-GTPs are inhibited by their Ran GAPs, which are located in the cytoplasm, generating this distinct compartmentation of the Ran GEFs and Ran GAPs. The Ran-activation gradient between these two compartments is responsible for nuclear molecular exporting (Figure 1) [27,28]. Besides, more recent studies have demonstrated that Ran-activation gradient could be indispensable for nuclear import, since its disruption impedes the entrance of large proteins inside the nucleus [29]. Otherwise, Ran can also modulate the assembly of mitotic spindles that control chromosome spatial organization during cell division [30]. It has recently been claimed that Ran overexpression improves cancer aggressiveness by promoting tumor proliferation, progression and metastasis [31]. Therefore, the development of certain molecules that reduce its expression and activation in carcinogenic cells could prevent cell proliferation by disturbing mitotic spindle formation, leading to promote their death by apoptosis [31].

The outer mitochondrial membrane GTPase Miro, which contains four EF hands and two GTPase domains [32], enables the distribution of mitochondria within the cell due to its ability to associate with the kinesin heavy chain [33]. Besides, other studies have recently suggested that the Miro EF domains act as Ca^2+^ sensors, increasing the mitochondrial uptake of this ion [33].

**Figure 2:**
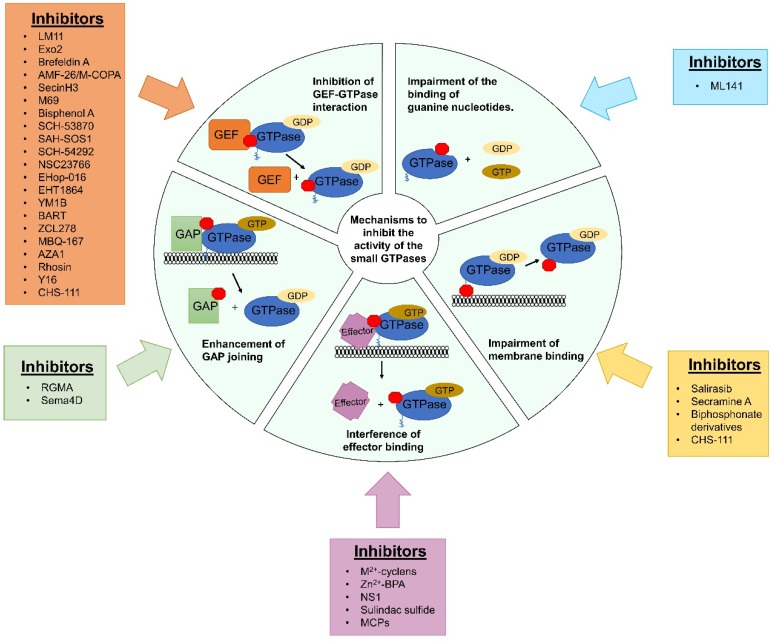
New strategies to target small GTPases in human cancers. To improve the therapeutic efficacy of inhibitors of small GTPases, new approaches have been developed by different strategies. Red boxes represent the inhibitor of GTPases in each of the strategies. Those include generation of new molecules that can fill the specific GEF binding site in GTPases, disruption of GEF-mediated guanine nucleotide exchange, filling of nucleotide binding pocket of small GTPases, impairing nucleotide attachment, and the stimulation of GAP proteins. Given that most of small GTPases need to be attached to the organelle membrane to exert their actions, the development of novel molecules with the ability to abolish this binding has arisen recently as an innovative strategy to inhibit these molecules. Finally, the development of some drugs that interfere with these could also be great to inhibit small GTPases. A brief table situated next to each section of the graphic indicates the small GTPase inhibitors that work through that mechanism.

Therefore, the development of small GTPase inhibitors could be a useful new treatment strategy for both non-carcinogenic and carcinogenic diseases [34]. However, the generation of these inhibitors is a challenging issue owing to the fine regulatory roles assigned to each of the members of the small GTPases protein family (Figure 2) [34,35]. Possible mechanisms by which new inhibitors can be designed include the development of molecules that prevent the formation of the specific GEF-GTPases complex, the impairment of the binding of GTP to GTPases, the increase of GAP protein activity to reduce the pool of active small GTPases, the blocking of the transduction of the activation signal to their specific downstream effector and the inhibition of their membrane-binding domain (Figure 2) [34,36].

## 2. Arf1 and Its Inhibitors in Cancer Therapy

Arf1 overexpression stimulates tumor progression and invasion, thus the inhibition of this protein could be useful for restraining cancer progression [12]. The inhibitors that are currently available to block this protein are listed in Table 1

For instance, LM11 can abolish Arf1 activation by inhibiting its association in the Golgi apparatus with its specific GEF, Arf nucleotide-binding site opener (ARNO) [37,38]. The high specificity of LM11 to Arf1 ensures that it does not abrogate other analogs, such as Arf6, making it suitable to treat Arf1-overexpressing tumors [37]. For instance, the treatment of breast tumors that overexpress Arf1 with LM11 reduces their aggressiveness by decreasing cell proliferation and invasion, as well as by inducing apoptosis [13,38]. Furthermore, LM11 disrupts breast cancer adhesion to the extracellular matrix by inhibiting paxillin translocation to the cell membrane [13], which is essential to connect integrins with the actin cytoskeleton [39]. Curiously, LM11 seems to be ineffective when tumor cells carry a K38A substitution in Arf1 [37], thus it is essential that Arf1 overexpressing tumors be tested for variants before the use of this inhibitor.

The Arf1 inhibitor Exo2 prevents the activation of certain Arf1-specific GEF enzymes by binding to their Sec7 domain [40]. This inhibition impedes the release of secretory vesicles from the endoplasmic reticulum (ER) to the Golgi apparatus [41,42], as well as reduces cellular lipid storage by inhibiting perilipin-2 expression [43]. Besides, this molecule presents high specificity for vesicle cargo since it impedes the retrograde transport of the Shiga toxin from the early endosomes to the ER, but does not hinder cholera toxin transport between the same compartments [42]. On the other hand, our research group reveals that Exo2 has the potential to reduce prostate tumor growth and metastasis through inhibiting Arf1-mediated ERK1/2 activation [44]. Most recently, we showed for the first time that active GTP-bound Arf1 is much higher in metastatic head and neck squamous cell carcinoma (HNSCC) cells compared with their paired non-metastatic cells, supporting the critical role of Arf1 activation in HNSCC metastasis [45]. We further provided evidence that EGF induces HNSCC cell invasion through the EGFR-Arf1 signaling complex and interrupting it using Exo2 or histone deacetylase inhibitor TSA deters the progression of HNSCC, providing a rational basis for Arf1-targeted anti-HNSCC therapy [45].

Brefeldin A (BFA) is a lactone-derived compound isolated from *Eupenicillium brefeldianum* that impairs Arf1 activation by hindering its association with its GEF enzyme [46,47]. The 7-hydroxyl residue of BFA seems to be essential to this process because its loss disrupts its affinity for the Arf1-GEF complex, preventing its inhibitory action [48]. This molecule can reduce anaplastic large cell lymphoma proliferation through reducing Arf1-dependent signal transducer and activator of transcription 3 (STAT3) phosphorylation [49]. It also presents a slight cytotoxic activity in other types of cancers, such as in lung, colorectal, ovarian, breast, prostate, melanoma or central nervous system [50]. Nevertheless, BFA shows poor bioavailability and high toxicity while exhibiting a number of pleiotropic effects in non-target organs, preventing the development of phase 1 clinical trials [42,49,51]. Therefore, the generation of new chemical derivatives of BFA with higher anticarcinogenic activity and lower off-target effects is essential to improve its use in cancer therapy [50,51]. For instance, acetylated BFA derivatives can reduce the viability of esophagus squamous cell carcinoma cells in a 500-times greater manner than native BFA [51]. Furthermore, ester BFA derivatives present higher potency than native BFA against different cancer types, which ultimately can reduce their off-target effects by lowering administered doses [50]. Finally, the addition of vinyl or aromatic groups to the C_15_ of BFA increases its ability to reduce HeLa cell proliferation [52].

AMF-26, also known as M-COPA, which was isolated from some species of the *Trichoderma* genus, also impairs the formation of the Arf1-GEF complex by disrupting GEF activity [47,53,54]. This molecule has greater bioavailability than BFA, increasing its feasibility for being used in cancer treatment [54]. In fact, AMF-26 can induce complete tumor regression in breast cancer xenografts [54], reduce the proliferation of 39 different cancers in a variety of human organs (such as breast, colon, kidney, skin, central nervous system, lung, ovary, and stomach) [53,55], as well as diminish angiogenesis through suppressing the activation of the vascular endothelial growth factor receptor 1/2 (VEGFR1/2) and the nuclear factor-κB (NF-κB) pathways [56]. In addition, AMF-26 deactivates a mutant form of the endolysosomal Kit, leading to sensitizing carcinogenic mast cell to imatinib [57]. Finally, AMF-26 also prevents Shiga toxin-dependent apoptosis by decreasing its translocation into the Golgi apparatus [58].

Sec7 inhibitor H3 (SecinH3) is a non-specific Arf inhibitor, which abrogates both Arf6 and Arf1 signaling by binding and inhibiting the Sec7 catalytic domain of ARNO and deactivating cytohesins, which are small ARF-specific GEFs [59,60]. SecinH3 was firstly developed to analyze the harmful effects that insulin resistance generates in human cells, since Arf6 down-regulation hinders insulin response in hepatic cells [60] and impairs glucose-stimulated insulin secretion in pancreatic β cells [61]. Moreover, this inhibitor can also reduce *Salmonella enterica* invasion by decreasing the Arf-activated pool [62]. Otherwise, this inhibitor presents great therapeutic effects in some carcinogenic diseases. For example, it diminishes the growth of breast xenografts and reduces breast-related lung metastasis and tumor aggressiveness [63]. Furthermore, it can also reduce the proliferation of certain non-small-cell lung cancer cell types by decreasing epithelial growth factor receptor (EGFR) activation and inducing apoptosis in both in vivo and in vitro models [64]. These beneficial effects ultimately reduce non-small-cell lung cancer resistance to gefitinib [64]. Finally, SecinH3 abolishes the migration, invasion, and proliferation of colorectal cancer cells in both in vivo and in vitro models [65].

M69, which is a RNA aptamer (an oligonucleotide that recognizes and attaches to a specific target with high affinity) [66], can impede Arf effects by deactivating GEF enzymes through binding to their catalytic Sec7 domain [67]. Although few experiments have been currently done with this inhibitor, it seems to present anti-carcinogenic effects as its expression in T lymphocytes leads to the reorganization of their actin cytoskeleton and to decreasing their adhesion to the extracellular matrix [67].

## 3. Ras and Its Inhibitors in Cancer Therapy

The RAS oncogenes (HRAS, NRAS and KRAS) comprise the most frequently mutated class of oncogenes in human cancers, stimulating intensive effort in developing anti-Ras inhibitors in order to get them to the clinic (Table 2). However, there is no effective Ras inhibitor to be used for cancer treatment [35,68]. Therefore, other chemical and biological strategies should be developed to accomplish the inhibition of this small GTPase.

### 3.1. Chemical Strategies for Suppressing Ras Activity

Ras has a flat tertiary structure, which does not provide clear sites where small repressing ligands can bind, apart from its nucleotide binding site [69]. The picomolar affinity of Ras for GTP allows for this GTPase to be activated when the concentration of this nucleotide is low [70]. These two properties are responsible for the clinical failure of most potential inhibitors for Ras [34]. New strategies are now focusing on the creation of an imbalance between Ras-specific GAP and GEF activities, as well as on the prevention of Ras from transducing its downstream proteins (Figure 2) [69]. Despite these two setbacks, recent in silico techniques have identified new potential inhibitor binding sites in the Ras molecule, opening the possibility for the development of more effective Ras inhibitors [69].

Two of these regions in Ras, which are designated as sites 1 and 2 and are located between the switch 1 domain and the second α-helix (H2) structure, are the regions where the most important Ras GEF enzyme, son of sevenless homolog 1 (SOS1), binds and activates Ras [69,71]. Therefore, the occupancy of these sites with small inhibitory molecules could impede Ras guanine nucleotide exchange, preventing the activation of its downstream effectors [69,71]. Screening of molecular libraries has identified new inhibitory compounds with promising oncostatic effects on many carcinogenic cell lines [72]. For example, bisphenol A and its derivative 4,4’-biphenol can inhibit the proliferation of NIH3T3 cells [73] and some SCH-53870 derivatives can induce the death of NIH3T3 cell through the disruption of Ras and SOS binding [74]. The development of a synthetic α-helical structure which mimics SOS α helix and sequesters Ras-GDP molecules has been reported to be a possible new strategy for the treatment of Ras-overexpressing tumors [71]. Finally, some SAH-SOS1 inhibitors have been shown to be able to restrain the viability of Ras-overexpressing pancreatic, colon and lung cancer cells, demonstrating their potential use in cancer therapy [75].

Site 3, which is located between the switch 2 domain and the third α-helix of Ras, also corresponds to a GEF binding site [69]. SCH-54292, one of the first developed Ras inhibitors, binds this region in an inverse Mg^2+^ concentration-dependent manner [76,77] and can restrain the growth of NIH3T3 cells by inducing apoptosis [78].

Sites 4 and 5 are located near the GTP-binding site and loop 7 of Ras and are the preferential joining site for divalent metal-cyclens (M^2+^-cyclens) [69,79]. M^2+^-cyclens are constituted by a divalent metallic ion (such as Zn^2+^, Co^2+^ or Cu^2+^) attached to an organic cyclic structure [80,81]. These compounds can stabilize Ras-GTP in a pre-activated state, which is also known as state 1(T) [79]. The 1(T) intermediate displays lower affinity for its downstream effectors, as opposed to Ras-GTP in the state 2(T) conformation, which is able to effectively activate its effector molecules [79]. Therefore, the induction of Ras-GTP into state 1(T) by M^2+^-cyclens inhibits the Ras-related Raf activation pathway [79]. More potent organometallic compounds have been developed, such as Zn^2+^-bis (2-picolyl) amine (Zn^2+^-BPA), which blocks the activation of the downstream effectors of Ras through its specific binding to the loop L7 and switch 1 domains of this protein [79]. Zn^2+^-BPA can even inhibit the activation of some mutated Ras analogs without the previous requirement of having Ras bound to GTP [79]. All these mentioned properties allow the potential use of these compounds in the treatment of Ras-overexpressing tumors [79].

Another target with potential oncostatic effects in Ras-overexpressing tumors is the interaction of Ras and Raf, which is an essential step in the transduction of Ras signal [69]. For example, a derivative from enantiomeric iridium (III) that blocks the interaction of Ras with Raf, hinders the proliferation of different cancer cell lines and reduces tumor volume in mice kidney xenografts by inhibiting the Ras-Raf dependent activation of the MAPK pathway [82]. Similarly, R11.1.6, a poly-β sheet protein, can also block Ras and Raf interaction, as well as MAPK pathway activation in embryonic kidney cells, also making it a promising molecule in targeting Ras-overexpressing cancer [83]. Although NS1, a promising synthetic monobody has not been proven in preclinical models yet, it is a strong Ras inhibitory molecule that prevents Ras dimerization and Ras–Raf interaction due to its ability to specifically bind to the α4 and α5 helices of Ras [70]. Another method to inhibit the Ras–Raf interaction is the use of specific non-steroidal anti-inflammatory drugs (NSAIDs) [84,85]. Sulindac sulfide is an NSAID that can reduce tumorigenesis as well as colorectal and breast cancer proliferation in vitro [84,85]. Sulindac sulfide interacts with Ras in a non-covalent manner, which ultimately reduces its ability to transduce downstream cellular signaling [84,85]. Besides, other NSAIDs, such as aspirin or indomethacin, can also inhibit Ras and Raf protein interaction in vivo [86]. Finally, some members of MCP family, such as MCP1, MCP53 and MCP110, can also abolish Ras and Raf interaction by directly binding to the Ras effector domain and indirectly controlling Ras and Raf folding [87]. More in detail, MCP110 can inhibit the growth of colorectal tumor xenografts and sensitize colon cancer cells to different chemotherapeutical drugs such as paclitaxel, docetaxel, vincristine, and sorafenib, supporting its use in the treatment of Ras-overexpressing tumors [88].

Another approach to control the activity of Ras includes the activation of Ras-specific GAPs [89]. This process would increase the level of inactive Ras protein and consequently decrease downstream Ras signal transduction [89]. However, some Ras mutants have been reported to be insensitive to this inhibitory strategy because of the lack of certain amino acids inside their GAP-binding domain, impeding the action of these enzymes [69]. Screening of molecular libraries has identified several inducers that activate Ras GAPs [89]. For instance, repulsive guidance molecule A (RGMA), which is a synthetic protein that can extend neuronal axons, dissociates p120GAP from FAK, enhancing p120GAP activity and ultimately reducing cellular Ras-GTP content [90]. Semaphorin 4D (Sema4D), which is implied in the growth of neuronal system, can restrain integrin-mediated cancer cell invasion and migration through inducing Ras-specific GAP Plexin-B1 via direct protein–protein interaction [89,91].

Finally, another strategy for Ras inhibition is the disruption of its anchorage to the cytoplasmic membrane, which is an essential step during its activation [92]. For instance, salirasib (also known as trans-farnesylthiosalicylic acid) can act as the carboxyl-terminal farnesylcysteine carboxymethyl ester of Ras, exhibiting sufficient inhibitory properties to be used in Ras-overexpressing cancer treatment [92,93]. Indeed, this inhibitor reduces dose-dependently the growth of pancreatic and lung cancer xenografts [94,95], and restrains dose- and time-dependently the proliferation of pancreatic and liver cancer cells in vitro through arresting cell cycle progression and stimulating apoptotic cell death [96,97]. Besides, salisarib potentiates the restraining effects of Exo2 on prostate cancer proliferation, invasion and migration through inducing apoptotic cell death in both in vitro and in vivo models [44]. This inhibitor also stimulates gemcitabine-dependent reduction of tumor volume and weight, leading to increasing the survival rate of mice with pancreatic tumor xenografts [94]. Nevertheless, it has been claimed that the normally administered doses of salirasib are insufficient to inhibit the growth of lung carcinomas that carry mutations in Ras [98], failing in most in vivo assays due to the compensatory action of geranylgeranyl transferases [34,99]. In conclusion, more studies are required to determine its efficacy in cancer treatment.

### 3.2. Non-Chemical Mechanisms for Suppressing Ras Activity

Because of the difficulty finding effective Ras inhibitors, other more indirect anti-Ras strategies have recently arisen, such as the induction of certain Ras-related lethal genes or the restoration of the metabolism disturbances induced by this small GTPase [35].

One of these approaches consists in the detection of some specific genes that are essential for the growth of Ras-mutated cells, but not for Ras-wild type cells [35]. Therefore, the knockdown of these genes leads to reducing the effect of Ras mutations in tumor cell proliferation and ultimately emerging as an effective strategy in the prevention of tumorigenesis, as well as enhancing the efficacy of Ras inhibitors [35,100]. Currently, a great number of synthetic lethal interactors have been identified in Ras-mutated cells through siRNA screening [100,101,102]. Some of these interactors are implicated in Ras maturation, such as prenyl protein-specific endoprotease 2 (*RCE1*), and protein-S-isoprenylcysteine O-methyltransferase (*ICMT*); while others are Ras effectors, such as *SHOC-2*, phosphatidylinositol-3,4,5-trisphosphate dependent Rac exchange factor 1 (*PREX1*) or *RAF1*; small GTPases, such as *RAC1*; or transcription factors, such as GATA-binding factor 1 (*GATA1*) [100,101,102]. However, the high differences existent among the different gene libraries makes this technique very inconsistent due to the high amount of false-negative results that occurs during the screening part [35]. Therefore, these protocols should be optimized to improve the sensitivity of this technique [35].

Another possible approach consists in the correction of the profound imbalances that the mutations in KRas generate in the metabolism of carcinogenic cells [35,103]. Indeed, KRas-mutated carcinogenic cells exhibit high levels of glucose uptake and glycolysis, fatty acid synthesis, glutaminolysis and nucleotide synthesis [103]. Due to the importance that these metabolic disturbances present in the maintenance of cancer promotion and progression, the methods that restore the metabolic rates to their basal levels could be an effective anti-cancer strategy [103]. In fact, tumorigenesis rate and pancreatic inflammation and fibrosis are clearly aggravated in mice carrying KRas tumors and are fed with high-fat diet [104]. The induction of autophagic response in KRas-mutated cells stimulates tumor aggressiveness and proliferation by accelerating glycolytic capability [105,106]. Indeed, autophagy inhibition in KRas-overexpressing tumors could decrease tumor promotion and progression [105,106].

## 4. Rac and Its Inhibitors in Cancer Therapy

Rac presents key functions in cancer promotion and progression since the aberrant expression of this small GTPase disrupts adherens cellular junctions, allowing cancer cells to undergo EMT and cell migration [21,107]. This alteration on Rac expression also enhances cellular proliferation by facilitating cell cycle progression from G1 to S phase and stimulating mitosis and cytokinesis [21,107]. Rac involvement in the reverse process of mesenchymal to epithelial transition (MET) facilitates the formation of secondary tumors [107]. These finding suggest that inhibition of Rac could be useful for cancer treatment [107]. The inhibitors that are currently available to block this protein, as well as their mechanism of action, are listed in Table 3

NSC23766 was one of the first developed Rac1 inhibitors with capability to discern from other Rho family GTPases, such as Cdc42 or RhoA [108]. This synthetic compound binds to a zone located between the switch I, switch II and β1/β2/β3 regions of this protein [108], impeding its activation by occupying the location where two RacGEF enzymes, Trio and T-lymphoma invasion and metastasis-inducing protein 1 (Tiam1) join together with this GTPase [108,109]. Due to the restrictive effects that present in Rac activation, NSC23766 has been proven to impede cell proliferation in both carcinogenic and non-carcinogenic cells [109]. For instance, this inhibitor can restrain prostate cancer proliferation and mobility, sensitize prostate cancer cells to ionizing radiotherapy, and inhibit non-small cell lung cancer (NSCLC) proliferation and invasion both in vivo and in vitro [109,110,111]. Inversely, this inhibitor was unable to prevent high-metastatic breast cancer cells migration, thus some new NSC23766 derivatives should be developed to improve its efficiency to restrain these types of cancers, as well as to reduce the administered doses [112].

One derivative of NSC23766, EHop-016, inhibits Rac activation through disrupting its direct binding to Vav2 (a Rho-specific GEF) [113], giving it a 100-fold lower IC_50_ against this GTPase than NSC23766, which encourages its use instead its derivative in cancer therapy [113,114,115,116]. In fact, EHop-016 can inhibit the proliferation of breast, myxofibrosarcoma and leukemic cancer cells in vitro, as well as impair their invasive capabilities [113,115,116]. It can also reduce the proliferation, angiogenesis and invasion of breast xenografts, diminishing the generation of secondary tumors in lung, liver, spleen and kidneys [114]. Besides, it reduces the growth of myxofibrosarcoma xenografts by restraining Rac-derived Akt/mTORC1 and mTORC2 activation [116]. Alternatively, this inhibitor has also been proven useful in the treatment of some non-carcinogenic disorders since it prevents glucose-induced insulin secretion and actin remodeling in pancreatic β cells [117].

EHT 1864 impedes the formation of the Rac-Tiam1 complex, keeping this GTPase in an inactive state [118]. This inhibitor is unable to inhibit other Rac-related Rho-family GTPases, demonstrating high specificity [118,119]. EHT 1864 exhibits promising potential properties to be used during cancer treatment [119,120]. For example, it can repress estrogen-induced breast cancer cell proliferation through modulating the Rac-dependent estrogen receptor-alpha (ERα) expression, as well as hampers NIH3T3 Rac-dependent Ras-induced tumorigenesis [119,120]. Besides, it prevents breast cancer invasion and proliferation, as well as inducing apoptosis through inhibiting STAT3 in patient-derived samples [121]. Finally, it can also sensitize breast carcinogenic cells to tamoxifen, since Rac1 overexpression seems to be a hallmark in the acquisition of cell resistance to this drug [120].

YM1B, which is a monoclonal antibody against CCN1, can halt breast cancer migration and invasion through inhibiting Rac induction and actin reorganization [122]. Besides, the generation of aptamers that inhibit Tiam1 activity, by impeding the binding of Rac1 to this molecule and decreasing its activation, is a new strategy to inactivation of this GTPase in the treatment of the tumors that overexpress Rac1 [123]. Conversely, binder of Arl Two (BART) abrogated carcinogenic cell mobility due to its capability to restrain the activation of this small GTPase [124]. Finally, core macrolactam and core macroketone, which are two migrastatin analogs, can also abolish breast cancer migration through inhibiting Rac activation [125].

Nevertheless, the constant use of Rac inhibitors in cancer treatment could generate some off-target effects in platelets because of the indirect inhibition of p21-activated kinase 1/2 (PAK1/2) [126], thus the administration of those Rac inhibitors should be controlled and interrupted when these undesired effects arise.

## 5. Cdc42 and Its Inhibitors in Cancer Therapy

Cdc42, which belongs to the Rho family [127], takes part in the initiation of most human cancers [21,128] since it induces the proliferation of Ras-transformed carcinogenic cells by inducing cell cycle progression [129], as well as stimulates tumorigenesis through impeding the degradation of EGFR by ubiquitin-proteasome system [128,130]. In addition, Cdc42 also enhances metastasis, invasion and EMT [127,131]. These properties back up the development of Cdc42 inhibitors as a great strategy to be used in the treatment of several malignant diseases. The inhibitors that are currently available to block this protein, as well as their mechanism of action, are listed in Table 3.

Secramine A, a synthetic derivate of galantamine, can disturb the recruitment of prenylated Cdc42 to the Golgi apparatus membrane by blocking Rho guanine dissociation inhibitor 1 (RhoGDI1), which modulates its attachment to this membrane [132,133]. Indeed, the inhibition of Cdc42 activity by this molecule generates a significant reduction in the proliferation of anaplastic large cell lymphoma cells through inducing apoptotic cell death [134]. Curiously, the lack of anaplastic lymphoma kinase (ALK) in these cells hinders the capability of secramine A to induce this phenotype, thus its utilization should be restrained to the cells that express this protein [134]. Otherwise, this inhibitor has also been shown to restrain other non-carcinogenic pathophysiological processes [135,136,137]. For example, secramine A alters fertilization process by reducing progesterone-induced and spontaneous acrosome reaction in mammalian sperm [135], reduces the propagation of human immunodeficiency virus (HIV) in cluster of differentiation 4 (CD4)^+^ T lymphocytes [136] and disturbs the release of collagen I from vascular smooth muscle cells to the cell matrix [137]. Nevertheless, some harmful off-target effects have been reported during the administration of this compound, limiting its use in carcinogenic and non-carcinogenic diseases [138].

Another potential inhibitor of Cdc42 with therapeutic applications in cancer is ZCL278, a 4-bromine-2-chlorophenol derivative that disrupts the joining between Cdc42 and intersectin (ITSN), which is a Cdc42-specific GEF enzyme, leading to inhibiting the activation of this small GTPase [139,140]. ZCL278 presents promising properties for being used in cancer therapy, such as a high membrane permeability and low toxicity for non-carcinogenic cells [137]. For example, ZCL278 can restrain the migratory and invasive characteristics of the prostate cancer cell line PC3 in vitro [139]. In addition, it can also prevent the toxicity of sodium arsenite (NaAsO_2_) on astrocytes and cerebellar granule neurons, encouraging its use to alleviate arsenic poisoning on the nervous system [141,142].

ML141 (also known as CID2950007 or CID2995007) and its analog CID44216842 are selective Cdc42 inhibitors that can deactivate this small GTPase in a non-competitive and allosteric manner by locking this protein in an inactive conformation [34,143]. Although these molecules are unable to induce any anti-proliferative effects in ovarian cancer cells, they can restrain dose-dependently their mobility, which indicates their possible use as a therapeutic adjuvants to reduce the arising of secondary tumors [138].

Additionally, some double Rac/Cdc42 inhibitors have been developed due to the similar roles these two small GTPases perform in cancer promotion and progression [144]. For instance, MBQ-167, which seems to inhibit the activation of Rac and Cdc42 by occupying their effector domain, prevents breast cancer cell migration, reduces cell viability, and impedes EMT progression by disrupting cell polarity without affecting non-carcinogenic cells growth (Table 3) [144]. N*2*,N*4*-Bis-(2-methyl-1H-indol-5-yl)-pyrimidine-2,4-diamine (AZA1), which is a structural derivative of NSC23766, can repress tumor growth and abolish cell migration in prostate xenografts (Table 3) [145]. Otherwise, R-ketorolac, which is an enantiomer of the analgesic S-ketorolac, can diminish cell adhesion to extracellular matrix and invasion in ovarian cancer cells, validating its possible use in cancer therapy [146].

## 6. Targeting other Small GTPases in Cancer Therapy

RhoA is an oncogene that induces tumor progression through enhancing carcinogenic cell proliferation, angiogenesis, invasion and metastasis [21,147], suggesting the development of RhoA inhibitors could be a great strategy to restrain cancer promotion and progression [36]. However, the generation of these inhibitors present several challenges due to the absence of stable cavities in the surface of RhoA, apart from the nucleotide-binding pocket, as well as its ability to capture guanine nucleotides at sub-nanomolar range [36]. Therefore, new strategies should be carried out to inhibit RhoA in cancer cells (Figure 2) [36]. The inhibitors that are currently available to block this oncogene, as well as their mechanism of action, are listed in Table 3.

Despite these difficulties, some promising RhoA inhibitors have already been developed to be used in cancer treatment [36]. For instance, Rhosin, which disturbs RhoA activation by binding to its W58 and impeding the docking of GEF enzymes [148], can inhibit breast cancer cell proliferation, migration and invasion in vitro and restrain the formation of spheroid bodies in gastric cancer cells [148,149]. On the other hand, Y16, which also prevents the binding between RhoA and its GEF enzymes by binding between the DH and PH domains of Leukemia-associated RhoGEF (LARG), suppresses breast cancer proliferation, migration and invasion, as well as modifies the distribution of the actin cytoskeleton [150]. Both inhibitors synergize to more efficiently hinder the interaction between RhoA and LARG, intensifying the reduction of breast cancer proliferation and invasion and allowing their use in the treatment of RhoA-overexpressing tumors [150].

CHS-111 is a benzyl indazole derivative that prevents both the joining of RhoA to membranes and its activation by its GEF enzyme Vav by preventing its interaction with phospholipase D1 (PLD1) [151]. Although there is no CHS-111 preclinical study, it might be useful in the treatment of RhoA overexpressing cancers.

Rab family constituents are also involved in the pathogenesis of some cancer types, such as hepatocellular carcinoma, cholangiohepatoma, gastric cancer, lung cancer, renal cancer, breast cancer, tongue and oral squamous cell carcinoma, as well as ovarian cancer [26,152,153,154,155,156,157], because they can promote cancer progression and aggressiveness by inducing invasion, metastasis, proliferation and cell cycle progression [26,157,158]. Rab protein overexpression also increases the resistance of carcinogenic cells to chemotherapy [159]. Despite the role of this family of small GTPases in cancer, few effective Rab inhibitors are available for cancer treatment [160]. Among them, Rab geranylgeranyltransferase (RabGGTase) inhibitors are the most promising molecules to be used in Rab-overexpressing tumors [160]. These inhibitors prevent the addition of geranyl groups in the C-termini of Rabs, which are necessary for ensuring their attachment to cellular membranes [160]. RabGGTases present promising effects in cancer therapy because they can effectively restrain human myeloma and mesothelioma cell proliferation in vitro by inducing apoptosis and cell cycle arrest, as well as reduce prostate and breast cancer adhesion and metastasis by suppressing the proteolytic activity of matrix metalloproteinases (MMPs) [161,162,163,164]. Therefore, we can conclude that RabGGTases show promising in vivo effects, preventing the growth of different skeletal tumors [165].

## 7. Conclusions

Small GTPases are upregulated in a broad spectrum of human cancers since they have the potential to promote cell proliferation and mobility as well as to stimulate their invasive and metastatic characteristics. However, no inhibitors are currently available for its normal use in clinical therapy. Indeed, small GTPases are difficult to target because they present few stable cavities for inhibitors to bind on their surface and they can capture guanine nucleotides at sub-nanomolar range. To achieve the inhibition of these molecules, some new strategies have recently arisen, such as the inhibition of the binding between GTPase and GEF, the enhancement of GAP activity, the hindering of nucleotide attachment, the blocking of their binding to cellular membranes, or the inhibition of the activity of their downstream effectors. However, most of the new drugs that hamper these strategies have only been proven successful in in vitro studies, while demonstrating fewer promising abilities at the in vivo model and clinical trial stages. These discrepancies normally occur because of the enhancement of some compensatory mechanisms that mask the effect of the inhibitor. Therefore, more in vivo studies should be performed to further evaluate the capability of these inhibitors to impede cancer progression and reduce cancer chemoresistance.

## Figures and Tables

**Table 1:** Action of Arf1 inhibitors in cancer treatment.

Name of the Inhibitor	Mechanism of Action	Model	Global Outcomes	Reference
LM11	Inhibition of ArfGEF binding to Arf1	Breast cancer cell lines cultured in vitro and breast cancer xenografts in zebrafish	Inhibition of cell proliferation, invasion and metastasis	[38]
		Breast adenocarcinoma cells cultured in vitro	Reduction of cell migration in a dose-dependent manner, cell adhesion to matrix and cell proliferation	[13]
Exo2	Inhibition of ArfGEF activity	Prostate cancer cells cultured in vitro	Suppression of cell proliferation, invasion and migration and induction of programmed cell death through apoptosis	[44]
BFA	Hindering of Arf1 and GEF joining	Anaplastic large cell carcinoma in vitro	Reduction of cell proliferation	[49]
		Lung, colon, melanoma, ovarian, renal, prostate, breast and central nervous system tumors in vitro	Increment of cell death and reduction of their proliferation	[50]
Acetylated BFA derivatives	Hindering of Arf1 and GEF joining	Esophagus squamous cell carcinoma in vitro	Increment of cell death in a sharper way than BFA	[51]
Ester derivatives of BFA	Hindering of Arf1 and GEF joining	Lung, colon, melanoma, ovarian, renal, prostate, breast and central nervous system tumors in vitro	Increment of cell death and reduction of their proliferation in a sharper way than BFA	[50]
C_15_ BFA derivatives	Hindering of Arf1 and GEF joining	Lung, colon, ovarian, renal, prostate, breast, leukemia, melanoma and central nervous system tumors in vitro	Increase of cell death, which is stronger than BFA	[52]
AMF-26/M-COPA	Impairment of ArfGEF activity	Breast cancer xenografts in vivo	Induction of complete reversion in the growth of these xenografts	[54]
		Melanoma cells both in vitro and in vivo models	Inhibition of angiogenesis, proliferation and tumor growth through the suppression of VEGFR1/2.	[56]
		Neoplastic mast cells cultured in vitro	Suppression of cell proliferation and resistance to imatinib through the abolishment of Kit signaling	[57]
SecinH3	Inhibition of ArfGEF binding to Arf1	Breast xenografts in vivo	Reduction of tumor growth, aggressiveness and metastasis	[63]
		Non-small cell lung cancer cell lines in vitro	Inhibition of cell proliferation and reduction of cell resistance to gefitinib	[64]
		Colorectal cancer models both in vivo and in vitro	Decrease cell proliferation, migration and proliferation through the abolishment of ARNO-dependent signaling	[65]
M69	Block of ArfGEF activity	Acute T cell leukemia cells cultured in vitro	Disturbance of intracellular adhesion through restructuration of actin skeleton	[67]

**Table 2:** Action of Ras inhibitors in cancer treatment.

Name of the Inhibitor	Mechanism of Action	Model	Global Outcomes	Reference
Bisphenol A	Disruption of the binding between Ras and SOS.	Cervical cancer cells cultured in vitro	Decrease in cell proliferation	[73]
SCH-53870 derivates	Disruption of the binding between Ras and SOS.	NIH3T3 mouse fibroblast in vitro	Decrease in cell proliferation both in normal and KRas-overexpressing cells	[74]
SAH-SOS1	Disruption of the binding between Ras and SOS.	Pancreatic, lung and colon cancer cells cultured in vitro bearing different KRAS mutants	Decrease in cell proliferation in a dose-dependent manner, independently of the KRAS mutant which bears the cells.	[75]
SCH-54292	Hindering of the binding between Ras and SOS	NIH3T3 mouse fibroblast in vitro	Inhibition of cell proliferation	[78]
MCP110	Inhibition of Raf and Ras-binding	Colon cancer models both in vivo and in vitro	Impediment of cell proliferation both in vitro and in vivo and synergy with other chemotherapeutic drugs, such as paclitaxel or vincristine	[88]
		Colon cancer cells cultured in vitro	Arrest of cell cycle in G_1_ phase through the abolishment of cyclin D1 levels	[87]
MCP1	Inhibition of Raf and Ras binding	Multiple myeloma cells cultured in vitro	Reduction of cancer cell growth through the induction of intrinsic apoptosis	[87]
MCP1 and MCP110	Inhibition of Raf and Ras binding	Multiple cancer cell lines defined by the National Cancer Institute (NCI) (Weinstein et al., 1997)	Reduction of cell proliferation	[87]
Enantiomeric iridium(III) metal-based compound	Inhibition of Ras and Raf interaction	Human kidney xenografts in vivo and kidney, breast, lung, prostatic, ovarian, melanoma and erythroleukemic cancer cell lines in vitro	Inhibition of cell cancer proliferation and reduction of tumor volume without affecting mice global weight	[83]
Sulindac sulfide	Hindering of Raf activation by Ras	NIH3T3 mouse fibroblast in vitro and Saos epithelial cells	Abolishment of Ras-dependent malignant transformation	[84]
		Brest cancer cells in vitro	Inhibition of E2-derivated pro-proliferative outcomes	[85]
Sema4D	Stimulation of Ras-GAP activity	Adrenal gland phaeochromocytoma cells cultured in vitro	Reduction of cell migration through inhibition of β_1_ integrin activation	[91]
Salirasib	Inhibition of Ras anchorage to cytoplasmic membranes	Pancreatic cell xenografts in vivo	Inhibition of tumor growth dose-dependently and stimulation of gemcitabine antiproliferative effects	[94]
		Lung cancer models both in vivo and in vitro	Inhibition of cell proliferation and tumor growth	[95]
		Hepatocellular carcinoma models both in vivo and in vitro	Inhibition of cell proliferation through the arrest of cell cycle and the induction of apoptosis	[96]
		Pancreatic cancer cells cultured in vitro	Reduction of cell proliferation through the arrest of cell cycle	[97]
		Prostate cancer cells cultured in vitro	Enhancement of Exo2 effects on cell proliferation, migration and invasion.	[44]
		Lung cancer patients	Common used doses and schedule failed in the inhibition of cell proliferation	[98]

**Table 3:** Action of Rho and Rab inhibitors in cancer treatment.

Name of the Inhibitor	Mechanism of Action	Model	Global Outcomes	Reference
NSC23766	Inhibition of RacGEF binding to Rac	Prostate cancer cells cultured in vitro	Reduction of cell proliferation and their invasive characteristics	[109]
		Pancreatic cancer cells in vitro	Increase of sensibility to radiotherapy	[111]
		NSCLC models both in vitro and in vivo	Inhibition of cell proliferation and migration. Increment of cell sensibility to gefitinib.	[110]
NSC23766 analogs	Inhibition of RacGEF binding to Rac	High-metastatic breast cancer cells cultured in vitro	Inhibition of cell proliferation in a sharper way than NSC23766 does	[112]
EHT 1864	Inhibition of RacGEF activity	Breast cancer cells cultured in vitro	Inhibition of cell proliferation stimulated by estrogen signaling	[120]
		Breast cancer cells cultured in vitro	Sensitization of cancer cells to tamoxifen	[120]
		NIH3T3 mouse fibroblast in vitro	Inhibition of Rac1-derived malignant cell transformation	[119]
		Breast cancer tumors biopsied from patients’ samples	Restraining of cell invasion and proliferation through programmed cell death induction	[121]
EHop-016	Inhibition of Vav1 and -2 activity and its binding with Rac	Metastatic breast cancer cells cultured in vitro	Reduction of cell viability and migration through the inhibition of Rac-derived actin structures	[113]
		Human and murine leukemic cell models both in vitro and in vivo and patient-derived cells	Increment of overall survival due to the inhibition of cell growth and survival	[115]
		Myxofibrosarcoma cell lines cultured in vitro and xenografts tumors cultured in vivo	Inhibition of cell growth through the induction of apoptosis and suppression of the generation of lung metastasis	[116]
		Breast cancer xenografts models with EHop-016 intraperitoneal administration	Repression of tumor growth, metastasis and angiogenesis	[114]
YM1B	Repression of RacGEF binding to Rac	Breast cancer cells cultured in vitro	Reduction of cell migration and invasion	[122]
BART	Repression of RacGEF activity	Pancreatic cancer cell lines cultured in vitro	Inhibition of cell motility and invasion through the regulation of actin cytoskeleton	[124]
Migrastatin analogs	Repression of Rac activity	High metastatic breast cancer cells in vivo xenograft models	Blockage of cell migration and metastasis through the inhibition of lamellipodia formation	[125]
Secramine A	Repression of Cdc42 shuttling between cytoplasm and cell membrane	ALCL cells cultured in vitro	Repression of cell proliferation through the induction of programmed cell death in ALK-positive cells	[133]
ZCL278	Inhibition of ITSN and Cdc42 binding	Prostate cancer cell lines cultured in vitro	Inhibition of cell motility and migration mediated by actin filaments	[139]
ML141 or CID2950007	Inhibition of GTP binding to Cdc42	Ovarian cancer cells cultured in vitro	Inhibition of cell motility and invasion without affecting to its viability	[138]
MBQ-167	Inhibition of GEF binding to Rac/Cdc42	Breast cancer cells cultured in vitro and xenografts models in vivo	Repression of cell migration, metastasis and proliferation	[144]
AZA1	Prevention of RacGEF binding to Cdc42/Rac	Prostatic cancer models both in vivo and in vitro	Decrease in cell proliferation through the induction of apoptosis in vitro. Reduction of tumor growth and improvement of mice survival in vivo	[145]
R-ketorolac	Inhibition of nucleotide docking	Ovarian cancer cell lines and primary patient-derived cells in vitro	Reduction in cell proliferation and growth	[146]
Rhosin	Inhibition of RhoAGEF binding to RhoA	Breast cancer cells cultured in vitro	Inhibition of cell proliferation, migration and invasion	[148]
		Diffuse gastric cancer spheroids cultured in vitro	Inhibition of cell proliferation, migration and invasion. Sensitization of cells to cisplatin	[149]
Y16	Hindering of RhoA and LARG joining	Breast cancer cells cultured in vitro	Reduction of cell proliferation and spheroid formation both alone and in combination with Rhosin	[150]
Biphosphonate derivatives	Inhibits Rab prenylation.	Melanoma cells cultured in vitro	Inhibition of cell proliferation through cell cycle arrest in S phase	[161]
		Mesothelioma cells cultured in vitro	Induction of cell apoptosis due to the inhibition of topoisomerase II and Rab6	[162]
		Prostate and breast cancer cell lines cultured in vitro	Inhibition of cell adhesion to extracellular matrix	[163]
		Prostate and breast cancer cell lines cultured in vitro	Inhibition of cell invasion and metastasis through the repression of MMPs activity	[164]

## Abbreviations

ALK	Anaplastic lymphoma kinase
ARL	Arf-like proteins
ARNO	Arf nucleotide-binding site opener
AZA1	N*2*,N*4*-Bis-(2-methyl-1H-indol-5-yl)-pyrimidine-2,4-diamine
BART	Binder of Arl Two
BFA	Brefeldin A
CD4	Cluster of differentiation 4
Cdc42	Cell division control protein 42 homolog
CERT	Ceramide transfer protein
EGFR	Epithelial growth factor receptor
EMT	Epithelial to mesenchymal transition
ER	Endoplasmic reticulum
ERK	Extracellular signal-regulated kinase
ERα	Estrogen receptor-alpha
Exo84	Exocyst complex component 84
FAK	Focal adhesion kinase
FAPP1	Phosphatidylinositol-four-phosphate adapter protein 1
GAP	GTPase-activating proteins
GATA1	GATA-binding factor 1
GEF	Guanine-nucleotide exchange factors
GRE	Rad and Gem-related proteins
H2	Second α-helix of Ras
HIV	Human immunodeficiency virus
HNSCC	Head and neck squamous cell carcinoma
IMCT	Protein-S-isoprenylcysteine O-methyltransferase
ITSN	Intersectin
LARG	Leukemia-associated RhoGEF
M^2+^-cyclens	Divalent metal-cyclens
M6PR	Mannose-6-phosphate receptor
MAPK	Mitogen-activated protein kinases
MET	Mesenchymal to epithelial transition
Miro	Mitochondrial Rho GTPase
MMP	Matrix metalloproteinase
mTORC1	mechanistic target of rapamycin complex 1
NF-κB	nuclear factor-κB
NSAID	Non-steroidal anti-inflammatory drug
NSCLC	Non-small cell lung cancer
p70S6K	Ribosomal protein S6 kinase beta-1
PAK	p21-activated kinase
PI3K	Phosphatidylinositol 3-kinase
PLCε	Phospholipase C epsilon
PLD1	Phospholipase D1
PREX1	Phosphatidylinositol-3,4,5-trisphosphate dependent Rac exchange factor
Rab	Ras-related in brain
RabGGTase	Rab geranylgeranyltransferase
Rac	Ras-related C3 botulinum toxin substrate
Rac1	Ras-related C3 botulinum toxin substrate 1
Rad	Ras-related associated with diabetes
Ral	Ras-like proteins
Ran	Ras-related nuclear protein
Rap	Ras-proximal proteins
RCE1	Prenyl protein-specific endoprotease 2
RGMA	Repulsive guidance molecule A
Rheb	Ras homolog enriched in brain
Rho	Ras-homolog
RhoA	Rho-related proteins
RhoBTB3	Rho-related BTB domain-containing protein 3
RhoGDI1	Rho guanine dissociation inhibitor 1
Rit	Ras-like in all tissues proteins
Rnd	Rho-related GTP-binding protein Rho6 precursor
RRAS	Ras-related proteins
SecinH3	Sec7 inhibitor H3
Sema4D	Semaphorin 4D
SNARE	NSF-attachment protein receptor
SOS1	Son of sevenless homolog 1
STAT3	Signal transducer and activator of transcription 3
TCL	TC10 and T-cell leukemia/lymphoma protein 1A
Tiam1	T-lymphoma invasion and metastasis-inducing protein 1
VEGFR	VEGF receptor
VEGF	Vascular endothelial growth factor
Zn^2+^-BPA	Zn^2+^-bis (2-picolyl) amine
